# Supplementary material for: Pathological changes in oral epithelium and the expression of SARS-CoV-2 entry receptors, ACE2 and furin
Source: PLoS One. 2024 Mar 15;19(3):e0300269. doi: 10.1371/journal.pone.0300269 (PMC10942036; doi:10.1371/journal.pone.0300269)
Supplement: S1 File — (PDF) [file pone.0300269.s001.pdf]

	group/diagnosis	furin total	furin basal	firin middle	furin upper
1	SCC	0	0	0	0
2	SCC	0	0	0	0
3	SCC	0	0	0	0
4	SCC	0	0	0	0
5	SCC	3	1	1	1
6	SCC	3	1	1	1
7	SCC	0	0	0	0
8	SCC	4.5	1.5	1.5	1.5
9	SCC	0	0	0	0
10	SCC	3	1	1	1
11	SCC	0	0	0	0
12	SCC	0	0	0	0
13	SCC	0	0	0	0
14	SCC	0	0	0	0
15	SCC	0	0	0	0
16	SCC	0	0	0	0
17	SCC	0	0	0	0
18	SCC	0	0	0	0
19	SCC	0	0	0	0
20	SCC	0	0	0	0
21	SCC	0	0	0	0
22	SCC	0	0	0	0
23	SCC	0	0	0	0
24	SCC	3	1	1	1
25	SCC	0	0	0	0
26	SCC	0	0	0	0
27	SCC	0	0	0	0
28	SCC	0	0	0	0
29	SCC	0	0	0	0
30	SCC	0	0	0	0
31	SCC	0	0	0	0
32	SCC	0	0	0	0
**33**	SCC	0	0	0	0
**34**	SCC	0	0	0	0
		**0.485294118**	**0.161764706**	**0.161764706**	**0.161764706**
		**1.191176471**	0.397058824	0.397058824	0.397058824
1	DYSP MOD	3	1	1	1
2	DYSP MOD	1	1	0	0
3	DYSP MOD	1	1	0	0
4	DYSP MOD	0	0	0	0
5	DYSP MOD	1	1	0	0
6	DYSP MOD	0	0	0	0
7	DYSP MOD	0	0	0	0
8	DYSP MOD	0	0	0	0
9	DYSP MOD	0	0	0	0
10	DYSP MOD	0	0	0	0
11	DYSP SEVERE	3	1	1	1
12	DYSP SEVERE	6	2	2	2
13	DYSP SEVERE	0	0	0	0

14	DYSP SEVERE	0	0	0	0
15	DYSP SEVERE	0	0	0	0
16	DYSP SEVERE	0	0	0	0
17	DYSP SEVERE	4	2	2	0
18	DYSP SEVERE	3	1	1	1
19	DYSP SEVERE	0	0	0	0
20	DYSP MOD	0	0	0	0
21	DYSP SEVERE	3	1.5	1.5	0
22	DYSP SEVERE	2	2	0	0
23	DYSP SEVERE	0	0	0	0
24	DYSP SEVERE	4.5	1.5	1.5	1.5
		**1.3125**	**0.625**	**0.416666667**	**0.270833333**
		1.754829645	0.739509973	0.687184271	0.558628653

1	DYSP MILD	1.5	1.5	0	0
2	DYSP MILD	0	0	0	0
3	DYSP MILD	0	0	0	0
4	DYSP MILD	4.5	1.5	1.5	1.5
5	DYSP MILD	1	1	0	0
6	HYP	4.5	1.5	1.5	1.5
7	HYP	0	0	0	0
8	HYP	0	0	0	0
9	HYP	0	0	0	0
10	HYP	3.5	1.5	1	1
11	HYP	1	1	0	0
12	HYP	3	1	1	1
13	HYP	3	1.5	1.5	0
14	HYP	3	1	1	1
15	HYP	1	1	0	0
16	HYP	1	1	0	0
18	HYP	3	1	1	1
19	HYP	3	1	1	1
20	HYP	0	0	0	0
21	HYP	0	0	0	0
22	HYP	0	0	0	0
23	HYP	1	1	0	0
24	HYP	0	0	0	0
25	HYP	0	0	0	0
26	HYP	0	0	0	0
27	HYP	0	0	0	0
		**1.307692308**	**0.634615385**	**0.365384615**	**0.307692308**
		1.52591034	0.613277643	0.564284644	0.520298048

1	NORMAL	0	0	0	0
2	NORMAL	0	0	0	0
3	NORMAL	0	0	0	0
4	NORMAL	0	0	0	0
5	NORMAL	1	1	0	0
6	NORMAL	0	0	0	0
7	NORMAL	0	0	0	0
8	NORMAL	0	0	0	0
9	NORMAL	0	0	0	0
10	NORMAL	0	0	0	0
11	NORMAL	0	0	0	0

12	NORMAL	0	0	0	0
13	NORMAL	0	0	0	0
14	NORMAL	1.5	1.5	0	0
		**0.178571429**	**0.178571429**	**0**	**0**
		0.447498717	0.447498717	0	0
1	OLP	3	1	1	1
2	OLP	0	0	0	0
3	OLP	1	1	0	0
4	OLP	0	0	0	0
5	OLP	0	0	0	0
6	OLP	1	1	0	0
7	OLP	1	1	0	0
8	OLP	0	0	0	0
9	OLP	4.5	1.5	1.5	1.5
10	OLP	0	0	0	0
11	OLP	0	0	0	0
12	OLP	0	0	0	0
13	OLP	1	1	0	0
14	OLP	0	0	0	0
15	OLP	1	1	0	0
16	OLP	1	1	0	0
17	OLP	0	0	0	0
18	OLP	1	1	0	0
19	OLP	0	0	0	0
20	OLP	3	1.5	1.5	0
21	OLP	3	1	1	1
22	OLP	0	0	0	0
23	OLP	1	1	0	0
24	OLP	3	1	1	1
25	OLP	1	1	0	0
26	OLP	4.5	1.5	1.5	1.5
27	OLP	0	0	0	0
28	OLP	0	0	0	0
29	OLP	0	0	0	0
30	OLP	0	0	0	0
31	OLP	0	0	0	0
32	OLP	0	0	0	0
33	OLP	0	0	0	0
34	OLP	0	0	0	0
35	OLP	0	0	0	0
36	OLP	1	1	0	0
37	OLP	0	0	0	0
38	OLP	1	1	0	0
39	OLP	0	0	0	0
40	OLP	0	0	0	0
41	OLP	0	0	0	0
42	OLP	0	0	0	0
43	OLP	0	0	0	0
44	OLP	0	0	0	0
45	OLP	0	0	0	0
46	OLP	0	0	0	0
47	OLP	0	0	0	0
48	OLP	0	0	0	0

49	OLP	0	0	0	0
50	OLP	0	0	0	0
51	OLP	0	0	0	0
		0.62745098	0.362745098	0.147058824	0.117647059
		1.14977891	0.524670124	0.411764706	0.364990401
